# Crystal structure and Hirshfeld surface analysis of 1-(2-chloro­acet­yl)-3-methyl-2,6-bis­(4-methylphen­yl)piperidin-4-one

**DOI:** 10.1107/S2056989026000083

**Published:** 2026-01-08

**Authors:** Sivagnanam Divyabharathi, Krishnan Rajeswari, Thankakan Vidhyasagar, Sivashanmugam Selvanayagam

**Affiliations:** a Department of Chemistry, Vel Tech Multi Tech Dr Rangarajan Dr Sakunthala Engineering College, Avadi, Chennai 600 062, India; b Department of Chemistry Annamalai University, Annamalainagar Chidambaram 608 002 India; c PG & Research Department of Chemistry, Government Arts College, Chidambaram 608 102, India; d PG & Research Department of Physics, Government Arts College, Melur 625 106, India; University of Neuchâtel, Switzerland

**Keywords:** piperidine derivative, inter­molecular hydrogen bonds, Hirshfeld surface analysis, crystal structure

## Abstract

In the crystal structure of the title compound, C_22_H_24_ClNO_2_, the piperidine ring adopts a boat conformation. Intra- and inter­molecular C—H⋯Cl hydrogen bonds are observed. The inter­molecular inter­actions were qu­anti­fied and analysed using Hirshfeld surface analysis, revealing that H⋯H inter­actions contribute most to the crystal packing (56.1%).

## Abstract 2

In the title compound, the crystal packing features C—H⋯Cl hydrogen bonds, which form *R*_2_^2^(12) graph-set motifs.

## 1. Chemical context

Piperidin-4-one derivatives represent a significant class of heterocyclic compounds widely documented for their versatility in the field of medicinal chemistry. The piperidin-4-one scaffold serves as a valuable synthetic inter­mediate and as a promising pharmacophore showing diverse biological activities (Sahu *et al.*, 2013 ▸). Among piperidin-4-one derivatives, 3-alkyl-2,6-di­aryl­piperidin-4-one derivatives have been extensively investigated, particularly with respect to their synthesis, stereochemistry, and diverse biological activities. 3-Alkyl-2,6-di­aryl­piperidone derivatives predominantly adopt a chair conformation with an equatorial orientation of the alkyl and phenyl substituents (Pandiarajan *et al.*, 1991 ▸). The introduction of groups such as –NO, –CHO, –COCH_3_, and N—COCH_2_Cl onto the ring nitro­gen atom of a 2,6-di­aryl­piperidin-4-one moiety significantly alter the ring conformation and the orientation of its substituents. Delocalization of the nitro­gen lone pair into the –CO*R* π-system imparts partial double-bond character to the —N—CO linkage, thereby restricting its rotation. The steric inter­action between the N—CO group and the neighbouring equatorial substituent causes mol­ecular strain, which is relieved by adopting a chair form with an axial orientation of the phenyl substituents or a boat form with one phenyl substituent in the flagpole position. The effects of such substitutions on the geometry of the piperidin-4-one nucleus have been extensively reported. Structural variations such as *N*-benzoyl (Krishnapillay *et al.*, 2000 ▸), *N*-nitroso (Ravindran *et al.*, 1991 ▸), *N*-formyl (Pandiarajan *et al.*, 1997 ▸), *N*-chloro­acetyl (Aridoss *et al.*, 2007*a* ▸,*b* ▸; Divyabharathi *et al.*, 2024 ▸) and *N*-thio­cyanato­acetyl (Karthiga *et al.*, 2024 ▸, 2025 ▸) derivatives have all been studied. Furthermore, investigations into the DNA-binding properties of *N*-acetyl analogues (Mohanraj & Ponnuswamy, 2018 ▸) and their anti­bacterial activities (Aridoss *et al.*, 2008 ▸) have also been reported. In the present work, crystal structure and Hirshfeld surface analysis of 1-(2-chloro­acet­yl)-3-methyl-2,6-bis­(4-methyl­phen­yl)piperidin-4-one, are reported.
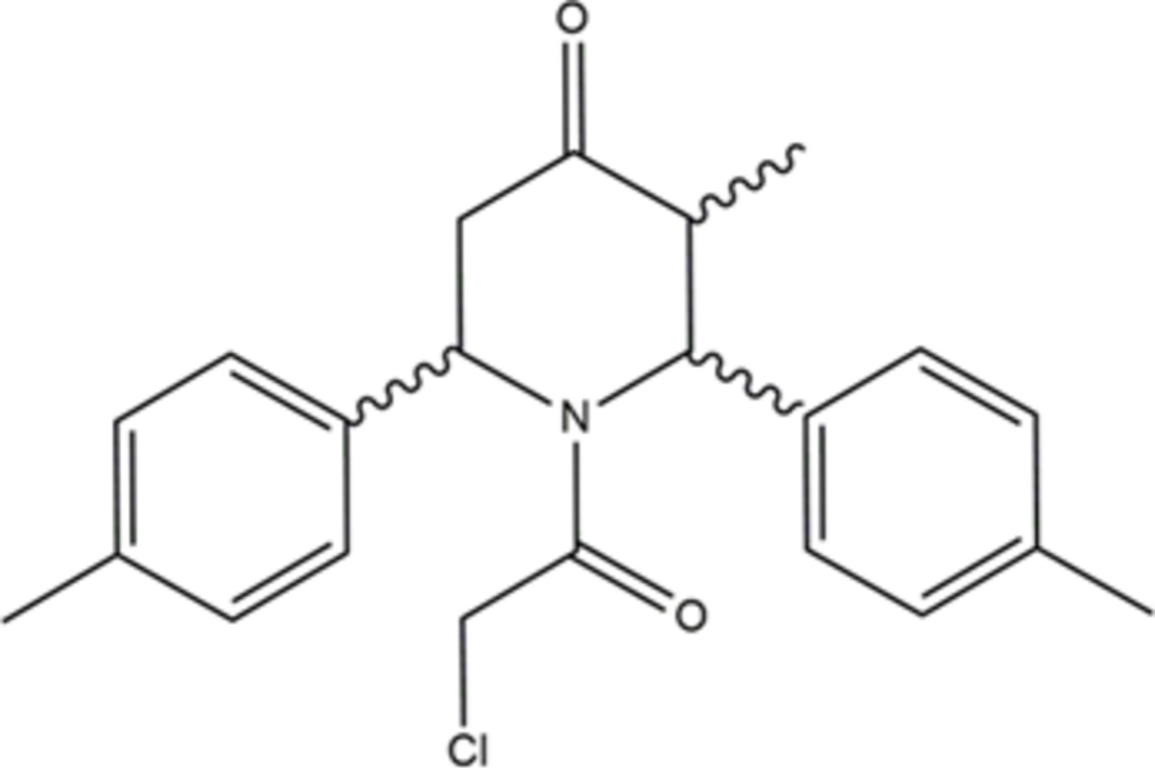


## 2. Structural commentary

**Figure 1:**
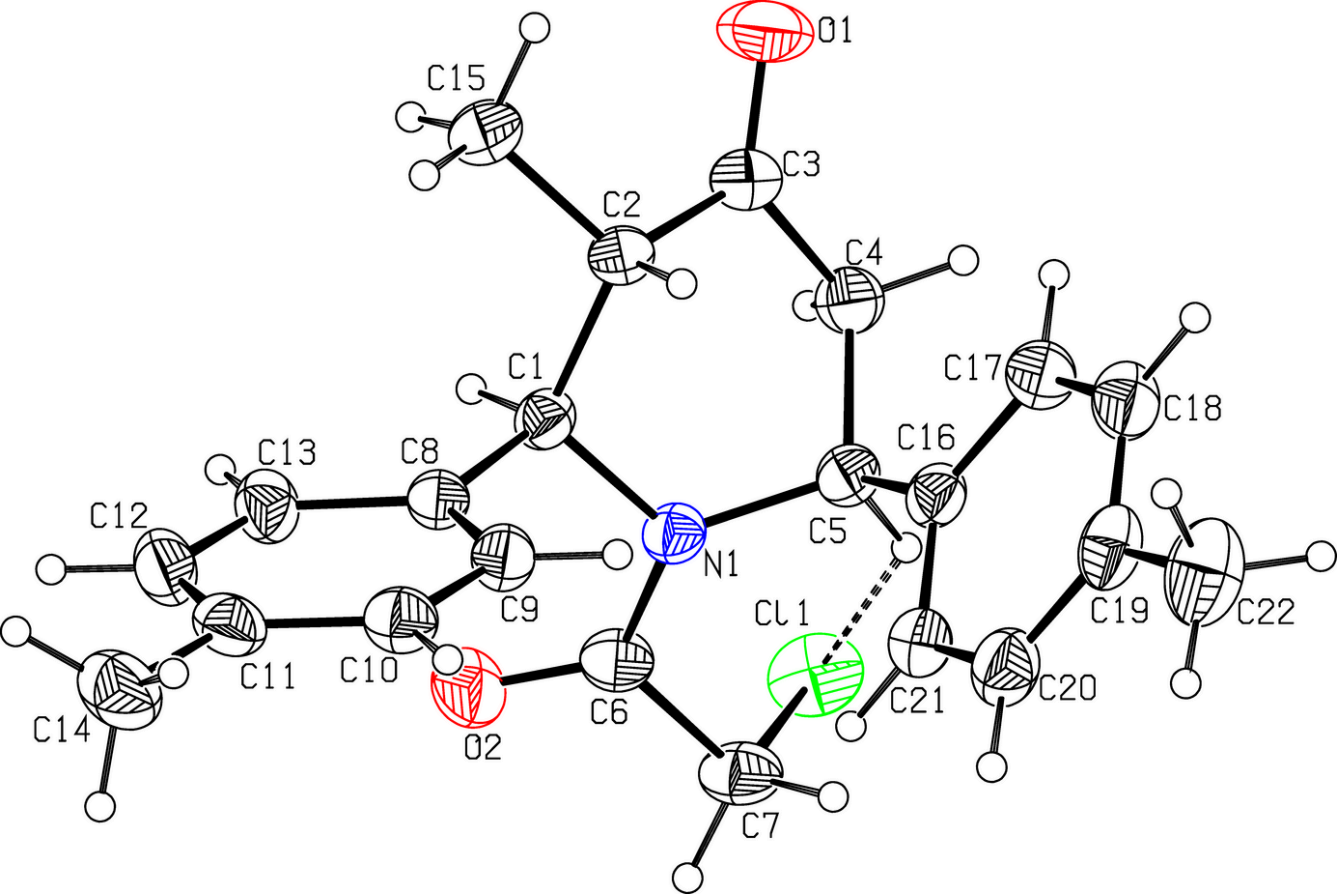
Mol­ecular structure showing the atom-labelling scheme and the intra­molecular hydrogen bond (dashed line). Ellipsoids are drawn at the 30% probability level.

The mol­ecular structure is presented in Fig. 1 ▸. The compound is chiral due to the presence of stereogenic centres. Although the mol­ecular structure depicted in Fig. 1 ▸ shows the 2*R*,3*S*,6*S* enanti­omer, the crystal contains a racemic mixture of enanti­omers. The O1—C3 [1.207 (2) Å] and O2—C6 [1.221 (2) Å] bond lengths confirm the double-bond character. The sum of the angles around atom N1 (357.1°) indicates that nitro­gen adopts an almost trigonal–planar geometry. Conjugation between the carbonyl group and the adjacent C—C bond, combined with steric hindrance from the chloro­methyl substituent, restricts free rotation about the C6—C7 bond. This limited rotational freedom results in distinct preferred conformations, which is reflected in the observed torsion angles O2—C6—C7—Cl1 [100.5 (2)°] and N1—C6—C7—Cl1 [−80.4 (2)°]. The piperidine ring adopts a boat conformation; the puckering parameters (Cremer & Pople, 1975 ▸) are: *q*_2_ = 0.677 (2) Å, *q*_3_ = −0.060 (2) Å, *Q*_T_ = 0.680 (2) Å and φ = 107.4 (2)°. Atoms C2 and C5 in the piperidine ring (N1/C1–C5) deviate by −0.528 (2) and −0.604 (2) Å, respectively, from the least-squares plane through the remaining four atoms. The methyl­phenyl rings C8–C13 and C15–C21 are planar, with their attached methyl atoms C14 and C22 deviate by −0.024 (3) and 0.003 (3) Å, respectively, from their ring planes. These methyl­phenyl rings are oriented with a dihedral angle of 51.7 (1)° with respect to each other. A weak intra­molecular contact (Table 1 ▸) between a methine H atom and the Cl atom attached to the 2-chloro­acetaldehyde moiety (C6/O2/C7/Cl1) leads to the stabilization of the mol­ecular conformation. This C5—H5⋯Cl1 inter­action forms an *S*(6) ring motif (Bernstein *et al.*, 1995 ▸), as shown in Fig. 1 ▸.

## 3. Supra­molecular features

**Figure 2:**
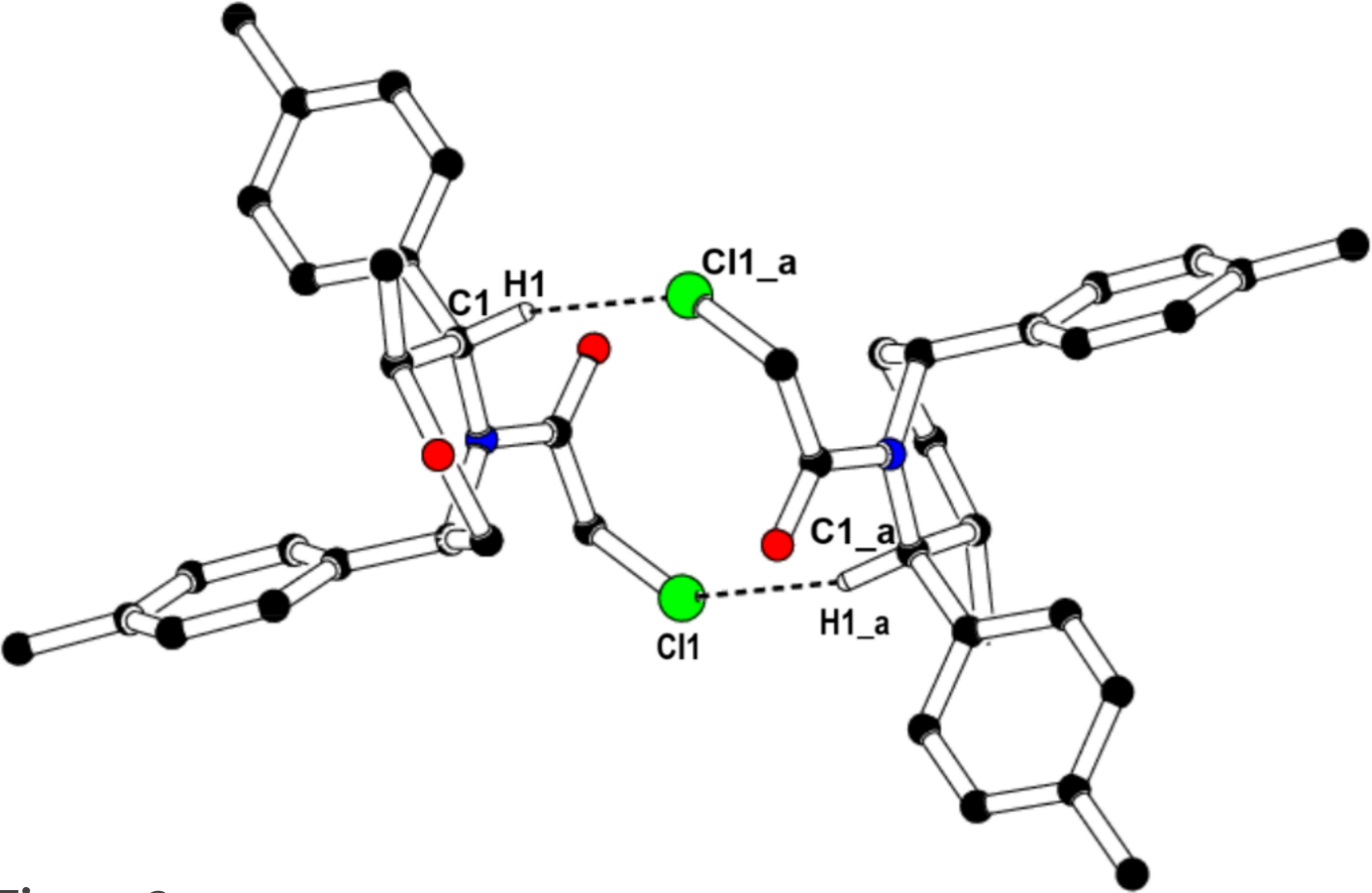
Centrosymmetric dimer through C—H⋯Cl hydrogen bonds [Symmetry code: (*a*) −*x* + 1, −*y*, −*z* + 1].

In the crystal, mol­ecules associate pairwise via C1—H1⋯Cl1^i^ hydrogen bonds (Table 1 ▸) into inversion dimers with an 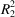
(12) graph-set motif (Etter *et al.*, 1990 ▸), as shown in Fig. 2 ▸. Moreover, mol­ecules are further linked into an 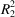
(14) graph-set motif by C—H⋯π inter­actions, C7—H7*A*⋯*Cg*, where *Cg* is the centroid of the symmetry-related C8–C13 benzene ring at (2 − *x*, 2 − *y*, 1 − *z*) (Table 1 ▸).

## 4. Hirshfeld surface analysis

**Figure 3:**
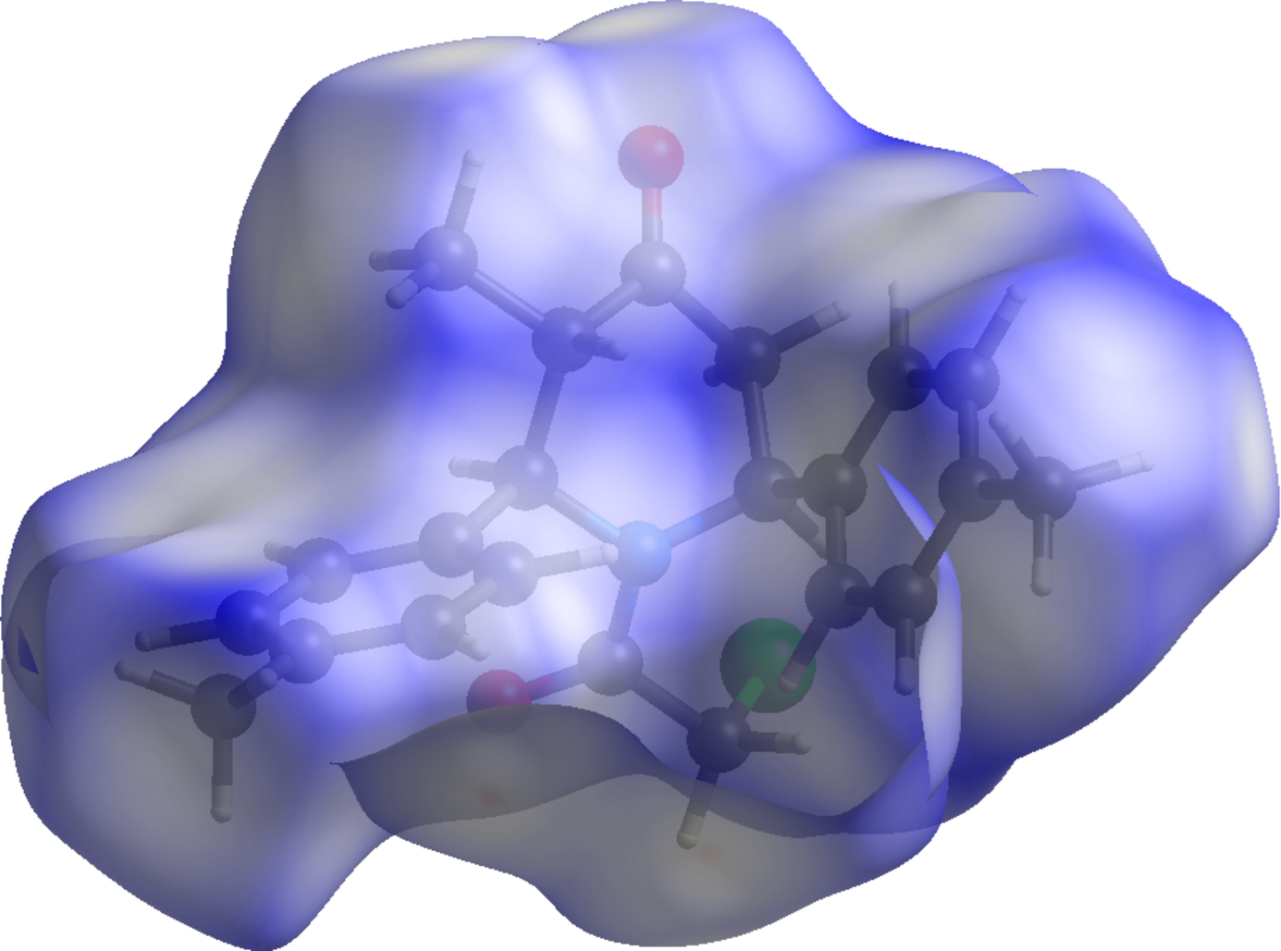
Hirshfeld surface mapped over *d*_norm._

**Figure 4:**
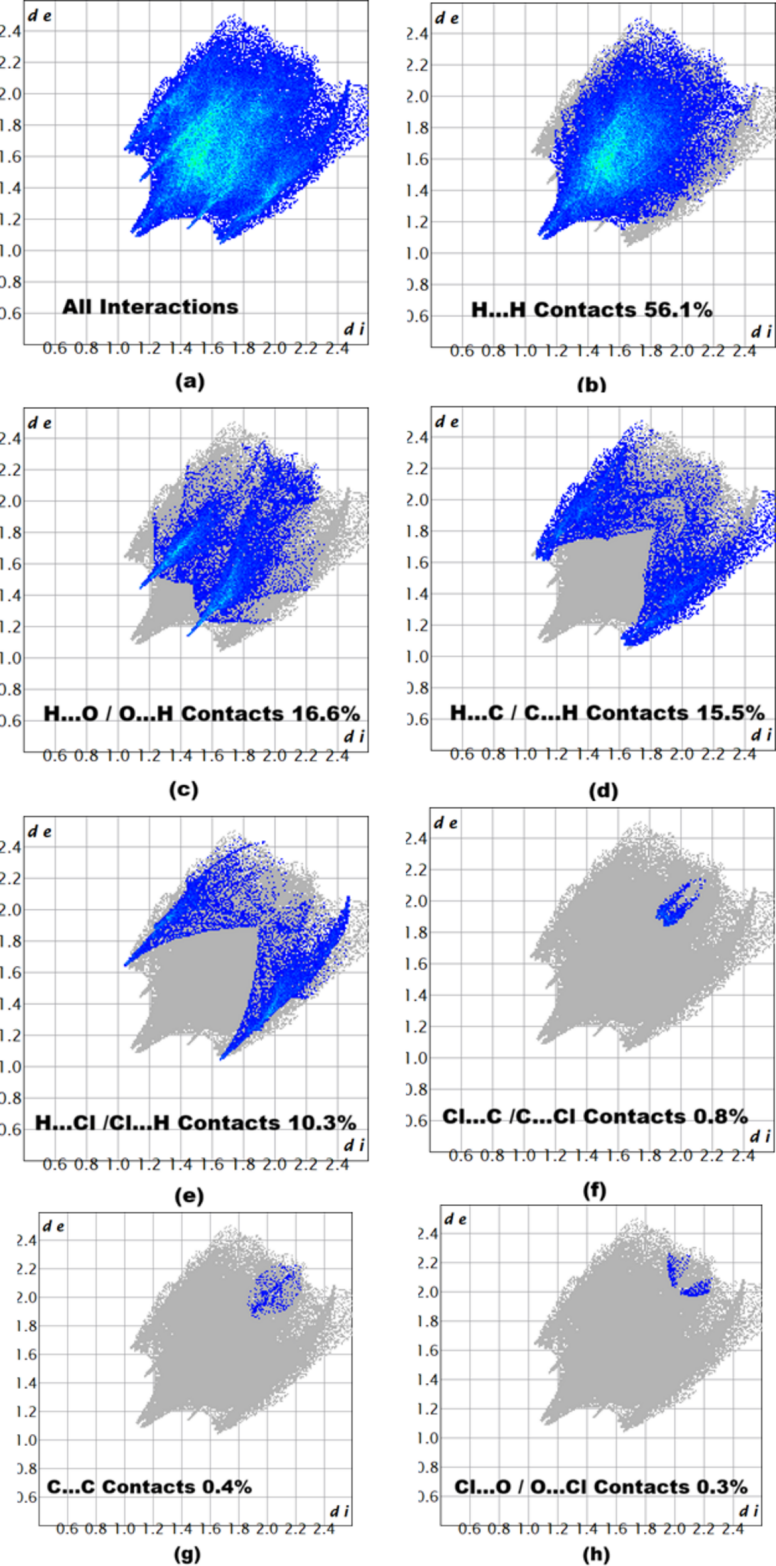
Two-dimensional fingerprint plots showing (*a*) all inter­actions, and delineated into (*b*) H⋯H, (*c*) H⋯O/O⋯H, (*d*) H⋯C/C⋯H, (*e*)H⋯Cl/Cl⋯H, (*f*) Cl⋯C/C⋯Cl, (*g*) C⋯C and (*h*) Cl⋯O/O⋯Cl inter­actions. The *d*_i_ and *d*_e_ values are the closest inter­nal and external distances (in Å) from given points on the Hirshfeld surface.

The inter­molecular inter­actions were qu­anti­fied by a Hirshfeld surface (HS) analysis (Spackman & Jayatilaka, 2009 ▸) using *CrystalExplorer* (Spackman *et al.*, 2021 ▸). The HS mapped over *d*_norm_ is illustrated in Fig. 3 ▸. where no red spot occurs. This represents the non-availability of potential hydrogen bonds in this crystal. The associated two-dimensional fingerprint plots (McKinnon *et al.*, 2007 ▸) provide qu­anti­tative information about the non-covalent inter­actions in the crystal packing in terms of the percentage contribution of the inter­atomic contacts (Spackman & McKinnon, 2002 ▸). The overall two-dimensional fingerprint plot is shown in Fig. 4 ▸*a* (top left). The HS analysis reveals that H⋯H and H⋯O/O⋯H contacts are the main contributors to the crystal packing followed by H⋯C/C⋯H, H⋯Cl/Cl⋯H, Cl⋯C/C⋯Cl, C⋯C and Cl⋯O/O⋯Cl contacts; see Fig. 4 ▸*b*–*h*.

## 5. Synthesis and crystallization

The compound has been previously reported, and all characterization data are consistent with those described by Aridoss *et al.*, 2007*a* ▸,*b* ▸. The compound was synthesized by mixing 3-methyl-2,6-di-*p*-tolyl­piperidin-4-one (0.75 g, 2.5 mmol) and chloro­acetyl chloride (1.0 mL, 10 mmol). The mixture was stirred in anhydrous benzene (50 mL) at room temperature. Then, tri­ethyl­amine (1.4 mL, 10 mmol) was added as a base to initiate the reaction. The reaction mixture was maintained at room temperature for 6 h. Upon completion, the precipitated tri­ethyl­ammonium chloride salt was removed by filtration. The resulting organic layer was washed thoroughly with water then dried over anhydrous Na_2_SO_4_. The solvent was removed and the crude product was recrystallized from a mixture of petroleum ether and ethyl acetate (9:1, *v*/*v*) to afford the product as colourless crystals.

## 6. Refinement

Crystal data, data collection and structure refinement details are summarized in Table 2 ▸. All H atoms were placed in idealized positions and allowed to ride on their parent atoms: C—H = 0.93–0.98 Å with *U*_iso_(H) = 1.5*U*_eq_(C) for methyl H atoms and *U*_iso_(H) = 1.2*U*_eq_(C) for other H atoms.

## Supplementary Material

Crystal structure: contains datablock(s) I, global. DOI: 10.1107/S2056989026000083/tx2106sup1.cif

Structure factors: contains datablock(s) I. DOI: 10.1107/S2056989026000083/tx2106Isup2.hkl

Supporting information file. DOI: 10.1107/S2056989026000083/tx2106sup3.txt

CCDC reference: 2184416

Additional supporting information:  crystallographic information; 3D view; checkCIF report

## Figures and Tables

**Table 1:** Hydrogen-bond geometry (Å, °) *Cg* is the centroid of the C8–C13 ring.

*D*—H⋯*A*	*D*—H	H⋯*A*	*D*⋯*A*	*D*—H⋯*A*
C5—H5⋯Cl1	0.98	2.61	3.342 (2)	132
C1—H1⋯Cl1^i^	0.98	2.79	3.674 (2)	151
C7—H7*A*⋯*Cg*^ii^	0.97	2.85	3.575 (2)	133

Symmetry codes: (i) 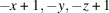; (ii) 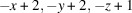.

**Table 2:** Experimental details

Crystal data	
Chemical formula	C_22_H_24_ClNO_2_
*M*_r_	369.87
Crystal system, space group	Triclinic, *P* 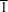
Temperature (K)	298
*a*, *b*, *c* (Å)	8.7654 (5), 11.3919 (6), 11.6090 (7)
α, β, γ (°)	110.594 (2), 102.709 (2), 107.595 (2)
*V* (Å^3^)	962.71 (10)
*Z*	2
Radiation type	Mo *K*α
μ (mm^−1^)	0.21
Crystal size (mm)	0.35 × 0.23 × 0.19

Data collection	
Diffractometer	Bruker APEXII CCD
Absorption correction	Multi-scan (*SADABS*; Krause *et al.*, 2015 ▸)
*T*_min_, *T*_max_	0.711, 0.746
No. of measured, independent and observed [*I* > 2σ(*I*)] reflections	24328, 5355, 3183
*R*_int_	0.043
(sin θ/λ)_max_ (Å^−1^)	0.706

Refinement	
*R*[*F*^2^ > 2σ(*F*^2^)], *wR*(*F*^2^), *S*	0.050, 0.135, 1.03
No. of reflections	5355
No. of parameters	238
H-atom treatment	H-atom parameters constrained
Δρ_max_, Δρ_min_ (e Å^−3^)	0.18, −0.27

Computer programs: *APEX3* and *SAINT* (Bruker, 2017 ▸), *SHELXT2018/2* (Sheldrick, 2015*a* ▸), *SHELXL2019/2* (Sheldrick, 2015*b* ▸), *ORTEP-3 for Windows* (Farrugia, 2012 ▸) and *PLATON* (Spek, 2020 ▸).

## supplementary crystallographic information

### 1-(2-Chloroacetyl)-3-methyl-2,6-bis(4-methylphenyl)piperidin-4-one Crystal data

**Table:**

C_22_H_24_ClNO_2_	*Z* = 2
*M**_r_* = 369.87	*F*(000) = 392
Triclinic, *P*1	*D*_x_ = 1.276 Mg m^−^^3^
*a* = 8.7654 (5) Å	Mo *K*α radiation, λ = 0.71073 Å
*b* = 11.3919 (6) Å	Cell parameters from 6744 reflections
*c* = 11.6090 (7) Å	θ = 2.6–28.8°
α = 110.594 (2)°	µ = 0.21 mm^−^^1^
β = 102.709 (2)°	*T* = 298 K
γ = 107.595 (2)°	Block, colourless
*V* = 962.71 (10) Å^3^	0.35 × 0.23 × 0.19 mm

### 1-(2-Chloroacetyl)-3-methyl-2,6-bis(4-methylphenyl)piperidin-4-one Data collection

**Table:**

Bruker APEXII CCD diffractometer	3183 reflections with *I* > 2σ(*I*)
Radiation source: i-mu-s microfocus source	*R*_int_ = 0.043
φ and ω scans	θ_max_ = 30.1°, θ_min_ = 2.1°
Absorption correction: multi-scan (SADABS; Krause *et al.*, 2015)	*h* = −11→12
*T*_min_ = 0.711, *T*_max_ = 0.746	*k* = −15→16
24328 measured reflections	*l* = −15→16
5355 independent reflections	

### 1-(2-Chloroacetyl)-3-methyl-2,6-bis(4-methylphenyl)piperidin-4-one Refinement

**Table:**

Refinement on *F*^2^	0 restraints
Least-squares matrix: full	Hydrogen site location: inferred from neighbouring sites
*R*[*F*^2^ > 2σ(*F*^2^)] = 0.050	H-atom parameters constrained
*wR*(*F*^2^) = 0.135	*w* = 1/[σ^2^(*F*_o_^2^) + (0.0437*P*)^2^ + 0.2977*P*] where *P* = (*F*_o_^2^ + 2*F*_c_^2^)/3
*S* = 1.03	(Δ/σ)_max_ < 0.001
5355 reflections	Δρ_max_ = 0.18 e Å^−^^3^
238 parameters	Δρ_min_ = −0.27 e Å^−^^3^

### 1-(2-Chloroacetyl)-3-methyl-2,6-bis(4-methylphenyl)piperidin-4-one Special details

**Table:**

Geometry. All esds (except the esd in the dihedral angle between two l.s. planes) are estimated using the full covariance matrix. The cell esds are taken into account individually in the estimation of esds in distances, angles and torsion angles; correlations between esds in cell parameters are only used when they are defined by crystal symmetry. An approximate (isotropic) treatment of cell esds is used for estimating esds involving l.s. planes.

### 1-(2-Chloroacetyl)-3-methyl-2,6-bis(4-methylphenyl)piperidin-4-one Fractional atomic coordinates and isotropic or equivalent isotropic displacement parameters (Å^2^)

**Table:**

	*x*	*y*	*z*	*U*_iso_*/*U*_eq_	
Cl1	0.25775 (8)	−0.21845 (5)	0.47830 (6)	0.0760 (2)	
O1	0.85771 (18)	0.29415 (17)	0.90454 (19)	0.0905 (6)	
O2	0.20241 (19)	0.06629 (14)	0.46708 (13)	0.0676 (4)	
N1	0.34459 (17)	0.11084 (13)	0.67584 (13)	0.0436 (3)	
C1	0.4496 (2)	0.25412 (16)	0.70160 (16)	0.0430 (4)	
H1	0.493746	0.246576	0.629971	0.052*	
C2	0.6072 (2)	0.33108 (17)	0.83254 (17)	0.0467 (4)	
H2	0.563298	0.350761	0.904614	0.056*	
C3	0.7039 (2)	0.2449 (2)	0.84836 (19)	0.0544 (4)	
C4	0.5966 (2)	0.09311 (18)	0.79297 (19)	0.0523 (4)	
H4A	0.639400	0.060054	0.854009	0.063*	
H4B	0.608122	0.044809	0.710194	0.063*	
C5	0.4065 (2)	0.05969 (17)	0.76895 (16)	0.0459 (4)	
H5	0.345337	−0.041045	0.722924	0.055*	
C6	0.2292 (2)	0.02538 (18)	0.55034 (17)	0.0498 (4)	
C7	0.1282 (2)	−0.12542 (18)	0.5127 (2)	0.0589 (5)	
H7A	0.025083	−0.164551	0.435285	0.071*	
H7B	0.093917	−0.133089	0.584595	0.071*	
C8	0.3460 (2)	0.33884 (16)	0.69974 (16)	0.0436 (4)	
C9	0.2647 (2)	0.36976 (18)	0.78849 (18)	0.0501 (4)	
H9	0.266432	0.332410	0.848017	0.060*	
C10	0.1809 (2)	0.45532 (19)	0.79026 (19)	0.0551 (5)	
H10	0.125999	0.473644	0.850156	0.066*	
C11	0.1777 (2)	0.51415 (19)	0.70398 (19)	0.0577 (5)	
C12	0.2585 (3)	0.4826 (2)	0.6152 (2)	0.0632 (5)	
H12	0.257599	0.520543	0.556190	0.076*	
C13	0.3407 (3)	0.39610 (19)	0.61200 (17)	0.0552 (5)	
H13	0.392961	0.375967	0.550488	0.066*	
C14	0.0891 (3)	0.6093 (2)	0.7089 (3)	0.0801 (7)	
H14A	0.090380	0.653225	0.796661	0.120*	
H14B	−0.027693	0.557154	0.647625	0.120*	
H14C	0.148117	0.678073	0.685548	0.120*	
C15	0.7241 (3)	0.4697 (2)	0.8473 (2)	0.0629 (5)	
H15A	0.665406	0.529360	0.857365	0.094*	
H15B	0.753294	0.455989	0.769991	0.094*	
H15C	0.827119	0.511073	0.923769	0.094*	
C16	0.3612 (2)	0.10762 (17)	0.89173 (17)	0.0459 (4)	
C17	0.4756 (3)	0.15250 (19)	1.01738 (18)	0.0566 (5)	
H17	0.586631	0.158729	1.028921	0.068*	
C18	0.4274 (3)	0.1882 (2)	1.12598 (19)	0.0623 (5)	
H18	0.507013	0.218030	1.209176	0.075*	
C19	0.2642 (3)	0.18084 (19)	1.1142 (2)	0.0605 (5)	
C20	0.1502 (3)	0.1367 (2)	0.9891 (2)	0.0628 (5)	
H20	0.039547	0.131290	0.977987	0.075*	
C21	0.1972 (2)	0.1003 (2)	0.87960 (19)	0.0546 (5)	
H21	0.117321	0.070404	0.796475	0.066*	
C22	0.2150 (4)	0.2203 (3)	1.2342 (2)	0.0888 (8)	
H22A	0.095320	0.202382	1.207207	0.133*	
H22B	0.282704	0.316607	1.292575	0.133*	
H22C	0.235683	0.167058	1.279197	0.133*	

### 1-(2-Chloroacetyl)-3-methyl-2,6-bis(4-methylphenyl)piperidin-4-one Atomic displacement parameters (Å^2^)

**Table:**

	*U*^11^	*U*^22^	*U*^33^	*U*^12^	*U*^13^	*U*^23^
Cl1	0.0787 (4)	0.0539 (3)	0.0795 (4)	0.0288 (3)	0.0323 (3)	0.0103 (3)
O1	0.0434 (8)	0.0746 (10)	0.1260 (15)	0.0156 (8)	0.0014 (9)	0.0413 (10)
O2	0.0794 (10)	0.0584 (8)	0.0419 (7)	0.0190 (7)	0.0066 (7)	0.0156 (6)
N1	0.0441 (8)	0.0359 (7)	0.0393 (7)	0.0099 (6)	0.0108 (6)	0.0130 (6)
C1	0.0439 (9)	0.0388 (8)	0.0402 (9)	0.0113 (7)	0.0144 (7)	0.0168 (7)
C2	0.0419 (9)	0.0395 (9)	0.0453 (9)	0.0094 (7)	0.0092 (7)	0.0154 (7)
C3	0.0427 (10)	0.0543 (11)	0.0571 (11)	0.0145 (9)	0.0129 (8)	0.0234 (9)
C4	0.0499 (10)	0.0485 (10)	0.0582 (11)	0.0215 (8)	0.0182 (9)	0.0237 (9)
C5	0.0464 (9)	0.0366 (8)	0.0468 (9)	0.0126 (7)	0.0129 (8)	0.0170 (7)
C6	0.0468 (10)	0.0432 (9)	0.0425 (10)	0.0138 (8)	0.0104 (8)	0.0092 (8)
C7	0.0478 (10)	0.0428 (10)	0.0591 (11)	0.0111 (8)	0.0088 (9)	0.0073 (9)
C8	0.0431 (9)	0.0375 (8)	0.0372 (8)	0.0094 (7)	0.0073 (7)	0.0138 (7)
C9	0.0524 (10)	0.0475 (10)	0.0484 (10)	0.0181 (8)	0.0160 (8)	0.0231 (8)
C10	0.0506 (10)	0.0515 (10)	0.0549 (11)	0.0194 (9)	0.0157 (9)	0.0189 (9)
C11	0.0486 (10)	0.0469 (10)	0.0573 (12)	0.0149 (9)	0.0006 (9)	0.0175 (9)
C12	0.0713 (13)	0.0591 (12)	0.0531 (11)	0.0229 (11)	0.0084 (10)	0.0308 (10)
C13	0.0650 (12)	0.0543 (11)	0.0415 (10)	0.0214 (10)	0.0146 (9)	0.0224 (8)
C14	0.0723 (15)	0.0693 (14)	0.0925 (17)	0.0371 (12)	0.0105 (13)	0.0352 (13)
C15	0.0533 (11)	0.0474 (10)	0.0654 (13)	0.0044 (9)	0.0095 (9)	0.0230 (10)
C16	0.0494 (10)	0.0372 (8)	0.0466 (10)	0.0124 (7)	0.0155 (8)	0.0199 (7)
C17	0.0559 (11)	0.0534 (11)	0.0502 (11)	0.0165 (9)	0.0132 (9)	0.0215 (9)
C18	0.0768 (14)	0.0496 (11)	0.0446 (10)	0.0157 (10)	0.0128 (10)	0.0187 (9)
C19	0.0852 (15)	0.0444 (10)	0.0581 (12)	0.0258 (10)	0.0336 (11)	0.0264 (9)
C20	0.0664 (13)	0.0701 (13)	0.0721 (14)	0.0326 (11)	0.0370 (11)	0.0427 (11)
C21	0.0539 (11)	0.0593 (11)	0.0542 (11)	0.0202 (9)	0.0202 (9)	0.0319 (9)
C22	0.133 (2)	0.0797 (16)	0.0738 (16)	0.0489 (17)	0.0600 (16)	0.0382 (13)

### 1-(2-Chloroacetyl)-3-methyl-2,6-bis(4-methylphenyl)piperidin-4-one Geometric parameters (Å, º)

**Table:**

Cl1—C7	1.789 (2)	C10—H10	0.9300
O1—C3	1.207 (2)	C11—C12	1.381 (3)
O2—C6	1.221 (2)	C11—C14	1.505 (3)
N1—C6	1.361 (2)	C12—C13	1.380 (3)
N1—C5	1.476 (2)	C12—H12	0.9300
N1—C1	1.493 (2)	C13—H13	0.9300
C1—C8	1.514 (2)	C14—H14A	0.9600
C1—C2	1.547 (2)	C14—H14B	0.9600
C1—H1	0.9800	C14—H14C	0.9600
C2—C3	1.509 (3)	C15—H15A	0.9600
C2—C15	1.526 (2)	C15—H15B	0.9600
C2—H2	0.9800	C15—H15C	0.9600
C3—C4	1.502 (3)	C16—C17	1.384 (2)
C4—C5	1.528 (2)	C16—C21	1.387 (3)
C4—H4A	0.9700	C17—C18	1.382 (3)
C4—H4B	0.9700	C17—H17	0.9300
C5—C16	1.523 (2)	C18—C19	1.380 (3)
C5—H5	0.9800	C18—H18	0.9300
C6—C7	1.519 (3)	C19—C20	1.381 (3)
C7—H7A	0.9700	C19—C22	1.506 (3)
C7—H7B	0.9700	C20—C21	1.385 (3)
C8—C9	1.383 (2)	C20—H20	0.9300
C8—C13	1.389 (2)	C21—H21	0.9300
C9—C10	1.384 (3)	C22—H22A	0.9600
C9—H9	0.9300	C22—H22B	0.9600
C10—C11	1.387 (3)	C22—H22C	0.9600
			
C6—N1—C5	122.82 (14)	C11—C10—H10	119.5
C6—N1—C1	115.84 (14)	C12—C11—C10	117.69 (18)
C5—N1—C1	118.41 (13)	C12—C11—C14	122.0 (2)
N1—C1—C8	113.14 (13)	C10—C11—C14	120.4 (2)
N1—C1—C2	112.07 (13)	C13—C12—C11	121.62 (18)
C8—C1—C2	110.05 (13)	C13—C12—H12	119.2
N1—C1—H1	107.1	C11—C12—H12	119.2
C8—C1—H1	107.1	C12—C13—C8	120.64 (18)
C2—C1—H1	107.1	C12—C13—H13	119.7
C3—C2—C15	112.14 (15)	C8—C13—H13	119.7
C3—C2—C1	112.78 (14)	C11—C14—H14A	109.5
C15—C2—C1	110.77 (14)	C11—C14—H14B	109.5
C3—C2—H2	106.9	H14A—C14—H14B	109.5
C15—C2—H2	106.9	C11—C14—H14C	109.5
C1—C2—H2	106.9	H14A—C14—H14C	109.5
O1—C3—C4	121.39 (18)	H14B—C14—H14C	109.5
O1—C3—C2	122.60 (18)	C2—C15—H15A	109.5
C4—C3—C2	115.99 (15)	C2—C15—H15B	109.5
C3—C4—C5	112.45 (15)	H15A—C15—H15B	109.5
C3—C4—H4A	109.1	C2—C15—H15C	109.5
C5—C4—H4A	109.1	H15A—C15—H15C	109.5
C3—C4—H4B	109.1	H15B—C15—H15C	109.5
C5—C4—H4B	109.1	C17—C16—C21	117.32 (17)
H4A—C4—H4B	107.8	C17—C16—C5	122.49 (16)
N1—C5—C16	112.38 (14)	C21—C16—C5	120.10 (15)
N1—C5—C4	108.01 (14)	C18—C17—C16	121.07 (19)
C16—C5—C4	116.21 (14)	C18—C17—H17	119.5
N1—C5—H5	106.5	C16—C17—H17	119.5
C16—C5—H5	106.5	C19—C18—C17	121.76 (19)
C4—C5—H5	106.5	C19—C18—H18	119.1
O2—C6—N1	122.11 (16)	C17—C18—H18	119.1
O2—C6—C7	118.71 (16)	C18—C19—C20	117.24 (18)
N1—C6—C7	119.17 (17)	C18—C19—C22	120.7 (2)
C6—C7—Cl1	109.87 (13)	C20—C19—C22	122.1 (2)
C6—C7—H7A	109.7	C19—C20—C21	121.4 (2)
Cl1—C7—H7A	109.7	C19—C20—H20	119.3
C6—C7—H7B	109.7	C21—C20—H20	119.3
Cl1—C7—H7B	109.7	C20—C21—C16	121.20 (18)
H7A—C7—H7B	108.2	C20—C21—H21	119.4
C9—C8—C13	117.95 (16)	C16—C21—H21	119.4
C9—C8—C1	122.47 (15)	C19—C22—H22A	109.5
C13—C8—C1	119.44 (16)	C19—C22—H22B	109.5
C8—C9—C10	121.14 (17)	H22A—C22—H22B	109.5
C8—C9—H9	119.4	C19—C22—H22C	109.5
C10—C9—H9	119.4	H22A—C22—H22C	109.5
C9—C10—C11	120.95 (19)	H22B—C22—H22C	109.5
C9—C10—H10	119.5		
			
C6—N1—C1—C8	70.04 (18)	C2—C1—C8—C9	−63.9 (2)
C5—N1—C1—C8	−128.78 (15)	N1—C1—C8—C13	−122.15 (16)
C6—N1—C1—C2	−164.83 (15)	C2—C1—C8—C13	111.63 (17)
C5—N1—C1—C2	−3.6 (2)	C13—C8—C9—C10	−0.1 (3)
N1—C1—C2—C3	46.1 (2)	C1—C8—C9—C10	175.48 (16)
C8—C1—C2—C3	172.88 (14)	C8—C9—C10—C11	−0.8 (3)
N1—C1—C2—C15	172.67 (15)	C9—C10—C11—C12	1.0 (3)
C8—C1—C2—C15	−60.51 (19)	C9—C10—C11—C14	−178.77 (18)
C15—C2—C3—O1	21.0 (3)	C10—C11—C12—C13	−0.3 (3)
C1—C2—C3—O1	146.9 (2)	C14—C11—C12—C13	179.49 (19)
C15—C2—C3—C4	−160.61 (16)	C11—C12—C13—C8	−0.7 (3)
C1—C2—C3—C4	−34.7 (2)	C9—C8—C13—C12	0.9 (3)
O1—C3—C4—C5	160.7 (2)	C1—C8—C13—C12	−174.90 (17)
C2—C3—C4—C5	−17.7 (2)	N1—C5—C16—C17	−138.29 (17)
C6—N1—C5—C16	−118.60 (17)	C4—C5—C16—C17	−13.2 (2)
C1—N1—C5—C16	81.61 (17)	N1—C5—C16—C21	45.3 (2)
C6—N1—C5—C4	111.89 (17)	C4—C5—C16—C21	170.40 (16)
C1—N1—C5—C4	−47.90 (18)	C21—C16—C17—C18	0.1 (3)
C3—C4—C5—N1	58.85 (19)	C5—C16—C17—C18	−176.44 (16)
C3—C4—C5—C16	−68.5 (2)	C16—C17—C18—C19	0.0 (3)
C5—N1—C6—O2	−166.44 (17)	C17—C18—C19—C20	−0.3 (3)
C1—N1—C6—O2	−6.2 (3)	C17—C18—C19—C22	179.97 (19)
C5—N1—C6—C7	14.5 (2)	C18—C19—C20—C21	0.5 (3)
C1—N1—C6—C7	174.76 (15)	C22—C19—C20—C21	−179.78 (19)
O2—C6—C7—Cl1	100.48 (19)	C19—C20—C21—C16	−0.4 (3)
N1—C6—C7—Cl1	−80.42 (19)	C17—C16—C21—C20	0.1 (3)
N1—C1—C8—C9	62.3 (2)	C5—C16—C21—C20	176.72 (16)

### 1-(2-Chloroacetyl)-3-methyl-2,6-bis(4-methylphenyl)piperidin-4-one Hydrogen-bond geometry (Å, º)

*Cg* is the centroid of the C8–C13 ring.

**Table:**

*D*—H···*A*	*D*—H	H···*A*	*D*···*A*	*D*—H···*A*
C5—H5···Cl1	0.98	2.61	3.342 (2)	132
C1—H1···Cl1^i^	0.98	2.79	3.674 (2)	151
C7—H7*A*···*Cg*^ii^	0.97	2.85	3.575 (2)	133

Symmetry codes: (i) −*x*+1, −*y*, −*z*+1; (ii) −*x*+2, −*y*+2, −*z*+1.
